# Cerebral toxoplasmosis with neurological co-infection in people living with AIDS/HIV: results of a prospective cohort in São Paulo, Brazil

**DOI:** 10.1055/s-0042-1759758

**Published:** 2023-03-14

**Authors:** João Paulo Marochi Telles, José Ernesto Vidal

**Affiliations:** 1 Instituto de Infectologia Emílio Ribas, São Paulo SP, Brazil.; 2 Universidade de São Paulo, Faculdade de Medicina, Hospital das Clínicas, São Paulo SP, Brazil.

**Keywords:** HIV, Cerebral Toxoplasmosis, Coinfection, HIV, Toxoplasmose Cerebral, Coinfecção

## Abstract

**Background** Concomitant neurological diseases in people living with HIV/AIDS (PLWHA) is a challenging subject that has been insufficiently evaluated by prospective clinical studies. The goal of the present study was to identify the clinical characteristics and outcomes of PLWHA with cerebral toxoplasmosis and neurological co-infections.

**Methods** We conducted a prospective observational cohort study at a tertiary teaching center in São Paulo, Brazil, from January to July 2017. Hospitalized PLWHA aged ≥ 18 years with cerebral toxoplasmosis were consecutively enrolled. A standardized neurological examination was performed at admission and weekly until discharge or death. Diagnosis and treatment followed institutional routines; neuroradiology, molecular diagnosis, neurosurgery, and the intensive care unit (ICU) were available. The main outcomes were neurological coinfections and in-hospital death.

**Results** We included 44 (4.3%) cases among 1,032 hospitalized patients. The median age was 44 (interquartile range [IQR]: 35–50) years, and 50% (n = 22) of the patients were male. The median CD4+ T lymphocyte count was of 50 (IQR: 15–94) cells/mm ^3^ . Multiple lesions on computed tomography were present in 59% of the cases. Neurological coinfections were diagnosed in 20% (n = 9) of the cases, and cytomegalovirus was the most common etiology (encephalitis: n = 3; polyradiculopathy: n = 2). Longer hospital stays (30 versus 62 days; *p* = 0.021) and a higher rate of ICU admissions (14% versus 44%; *p* = 0.045) were observed among PLWHA with neurological coinfections in comparison to those without them. The rate of in-hospital mortality was of 13.6% (n = 6) (coinfection group: 33%; no coinfection group: 8.6%; *p* = 0.054).

**Conclusion** Neurological c-infections were common among PLWHA with cerebral toxoplasmosis, and cytomegalovirus was the main copathogen. The group of PLWHA with neurological co-infections underwent longer hospital stays and more frequent intensive care unit admissions. Additionally, this group of patients tended to have higher in-hospital mortality rate.

## Resumo

**Antecedentes** Coinfecções neurológicas em pessoas que vivem com HIV/AIDS (PVHA) é um tema que não foi suficientemente avaliado em estudos clínicos prospectivos. Nosso objetivo foi identificar as características clínicas e os desfechos de PVHA com toxoplasmose cerebral e coinfecções neurológicas.

**Métodos** Estudo prospectivo de coorte observacional conduzido em um centro de ensino terciário de São Paulo, Brasil, entre janeiro e julho de 2017. Foram incluídos consecutivamente PVHA internadas com ≥ 18 anos e toxoplasmose cerebral. Realizou-se exame neurológico padronizado na admissão e semanalmente até a alta/óbito. Tanto o diagnóstico quanto o tratamento seguiram a rotina institucional; neurorradiologia, diagnóstico molecular, neurocirurgia e Unidade de Terapia Intensiva (UTI) estavam disponíveis. Os principais desfechos foram coinfecções neurológicas e óbitos hospitalares.

**Resultados** Incluímos 44 (4,3%) casos entre 1.032 pacientes internados. A idade mediana foi de 44 (intervalo interquartil [IIQ]: : 35–50) anos, e 50% (n = 22) dos pacientes eram do sexo masculino. A contagem mediana de linfócitos T CD4+ foi de 50 (IIQ:15–94) células/mm ^3^ . Múltiplas lesões na tomografia computadorizada foram observadas em 59% dos casos. Coinfecções neurológicas foram diagnosticadas em 20% (n = 9) dos casos, sendo o citomegalovírus a etiologia mais comum (encefalite: n = 3; polirradiculopatia: n = 2). Observou-se maior tempo de internação (26 *versus* 62 dias; *p* = 0,021) e uma taxa mais alta de admissão à UTI (14% *versus* 44%; *p* = 0,045) em PVHA com coinfecções neurológicas em comparação àquelas sem coinfecção. A mortalidade intra-hospitalar foi de 13,6% (n = 6) (grupo com coinfecções: 33% *versus* grupo sem coinfecção: 8,6%; *p* = 0,054).

**Conclusão** Coinfecções neurológicas foram comuns em PVHA com toxoplasmose cerebral, sendo o citomegalovírus o principal copatógeno. O grupo de PVHA com coinfecções neurológicas apresentou maior tempo de internação, maior taxa de internações na UTI, e tendência a maior taxa de mortalidade intra-hospitalar.

## INTRODUCTION

Cerebral toxoplasmosis is the main neurological disease associated with people living with HIV/AIDS (PLWHA). 1 Despite its decreasing incidence in the era of the combined antiretroviral therapy (cART), cerebral toxoplasmosis remains an important cause of hospital admission in low- and middle-income countries. 2

The risk factors are well known, and include the prevalence of *Toxoplasma gondii* infection in the general population, low CD4+ T lymphocyte count, and a lack of anti- *T. gondii* prophylaxis. 3 Nevertheless, there are few studies on the outcomes of cerebral toxoplasmosis in PLWHA in the post-cART era, particularly in Latin America. 3

Concomitant neurological diseases in PLWHA are challenging to treat and have not been sufficiently evaluated in prospective clinical studies. As exposure to pathogens varies by region, the incidence of coinfection and other diseases may also be influenced by the current epidemiological scenario. For example, given the high prevalence of *Cryptococcus neoformans* and *Mycobacterium tuberculosis* in India, coinfections by these pathogens are more common in this region than in Europe or North America. 4 Suspicion and timely identification of concomitant neurological diseases in PLWHA enable the provision of the appropriate treatment for these patients. The present study aimed to identify the frequency, key features, and outcomes of cerebral toxoplasmosis with neurological coinfection in PLWHA.

## METHODS

### Setting

The present study was conducted at Instituto de Infectologia Emilio Ribas, a referral infectious disease hospital in the city of São Paulo, Brazil, between January 1 and August 31, 2017.

### Design

The present prospective observational cohort study on PLWHA experiencing cerebral toxoplasmosis and neurological coinfections is a sub-analysis of a study published elsewhere. 5

### Patients

The eligible patients had HIV/AIDS with neurological complaints and were admitted to hospital. The inclusion criteria were: age ≥ 18 years; confirmed HIV diagnosis; diagnosis of cerebral toxoplasmosis during hospitalization; and diagnosis of other neurological coinfections during hospitalization. Patients were excluded if they presented exacerbation of previous neurological sequelae. The study sample was followed up until death or discharge.

### Definitions

Cerebral toxoplasmosis was defined as confirmed or probable according to the following criteria: *confirmed* – positive histopathological or polymerase chain reaction (PCR) of the cerebrospinal fluid (CSF) of patients with clinical and radiological improvement after 10 to 14 days of treatment; and *probable* – compatible clinical and radiological findings with improvement after 10 to 14 days of treatment. Neurological coinfections were defined as confirmed or probable according to clinical, neuroradiological, and CSF testing (including basic characteristics, cultures, antigen tests, and molecular techniques), along with histopathological results. 5

### Variables

Sex, age, CD4+ T lymphocyte count, viral load, previous HIV medical history (opportunistic diseases and cART use), main and associated neurological symptoms, findings of neurological examinations, length of hospital stay, outcomes of hospitalization, and brain images obtained by computed tomography (CT) and/or magnetic resonance imaging (MRI) were recorded. Neurological diseases and etiological diagnoses during hospitalization were also documented.

### Measurements and outcomes

A neurological examination was performed on admission, and follow-up was continued weekly until death or discharge. Clinical, laboratory, and radiological data were prospectively collected. Diagnosis and treatment were based on institutional protocols, and the investigators did not influence the management of the cases. The history of CD4+ T lymphocyte count and viral load was collected using the National Network Laboratory Testing System of CD4 +/CD8+ T lymphocyte count and HIV viral load.

The outcomes of the present study included length of hospital stay, admission to the Intensive Care Unit (ICU), and in-hospital mortality in patients with concomitant neurological infections and in all patients with cerebral toxoplasmosis.

### Statistical analysis

A normality test was used to define the population distribution of the quantitative variables. Normally-distributed quantitative variables were analyzed with the Student *t* -test and non-normal data, with the Mann–Whitney U test. Chi-squared tests were used to analyze the qualitative variables. Variables with values of *p* < 0.2 were selected for binary logistic regression. Data were analyzed using Statistical Package for the Social Sciences (IBM SPSS Statistics for Windows, IBM Corp., Armonk, NY, US) software, version 22.0. Statistical significance was set at *p* < 0.05.

## RESULTS

### General results

A total of 1,032 patients were admitted; 44 (4.3%) of them had cerebral toxoplasmosis, and their clinical and radiological characteristics are presented in Table 1 . The median age was of 44 (interquartile range [IQR]: 35–50) years, and 22 patients (50%) were men. Only 3 (6.8%) subjects were diagnosed with HIV infection during hospitalization. A previous cART treatment was reported in 39 patients (88%). Other opportunistic diseases were diagnosed in 26 patients (59%) during hospitalization. The median CD4+ T lymphocyte count was of 50 (IQR: 15–94) cells/mm ^3^ .

**Table 1:** Epidemiological, clinical, and radiological characteristics of 44 patients living with HIV/AIDS and cerebral toxoplasmosis

General characteristics	
Age in years: median (IQR)	44 (35–50)
Male sex: n (%)	22 (50%)
Unknown HIV infection: n (%)	3 (6.81%)
Duration of HIV infection in years: median (IQR)	10 (3.4–17)
Previous opportunistic diseases: n (%)	26 (59%)
Anti- *Toxoplasma gondii* prophylaxis: n (%)	27 (61%)
History of cART: n (%)	39 (88%)
Protease inhibitors in cART: n (%)	15 (38%)
Integrase inhibitors in cART: n (%)	4 (10%)
CD4+ T lymphocytes in cells/mm ^3^ : median (IQR)	50 (15–94)
Viral load in copies/mm ^3^ : median (IQR)	81.290 (4.600–251.598)
**Clinical characteristics**	
Duration of symptoms before hospital admission in days: median (IQR)	10 (5–25)
Altered level of consciousness: n (%)	13 (29%)
Seizure: n (%)	12 (27%)
Hemiparesis: n (%)	20 (45%)
Headache: n (%)	27 (61%)
Sphincter dysfunction: n (%)	4 (9%)
**Neurological syndromes**	
Focal encephalitis: n (%)	27 (61%)
Diffuse encephalitis: n (%)	6 (13%)
Meningoencephalitis: n (%)	6 (13%)
Others neurological syndromes	4 (9%)
Anti- *T. gondii* IgG: n (%)	33 (75%)
Positive IgG: n (%)	32 (96%)
CSF PCR collected: n (%)	17 (38%)
Positive CSF PCR: n (%)	3 (17%)
Brain biopsy: n (%)	1 (2%)
Neurological coinfections: n (%)	9 (20%)
ICU admission: n (%)	8 (18%)
**Radiological characteristics**	
Computed tomography: n (%)	44 (100%)
Single focal lesion: n (%)	18 (41%)
Multiple focal lesions: n (%)	26 (59%)
**Outcomes**	
Death	6 (13%)

Abbreviations: cART, combined antiretroviral therapy; CSF, cerebrospinal fluid; IgG, immunoglobulin G; IQR, interquartile range; ICU, Intensive Care Unit; PCR, polymerase chain reaction.

The median duration of the neurological symptoms before hospital admission was of 10 (IQR: 5–25) days. The main neurological syndrome observed was focal encephalitis (n = 27; 61%); however, 12 patients (26%) presented with diffuse encephalitis or meningoencephalitis. Multiple focal brain lesions were observed on CT in 26 patients (59%), and 8 patients (18%) were referred to the ICU.

### Etiological diagnosis and neurological coinfections

Anti- *T. gondii* immunoglobulin G (IgG) and immunoglobulin M (IgM) antibodies were detected in 33 patients (75%). Positive IgG and IgM were observed in 32 (96%) and 1 (3%) of the patients respectively. The CSF PCR test was available for 17 (38%) patients, and it was only positive in 3 (17%). The median time from anti- *T. gondii* treatment to *T. gondii* sample collection for PCR was of 13 (IQR: 4–25) days. Cerebral toxoplasmosis was considered confirmed in 3 (7%) and probable in 41 (93%) patients. Cerebral biopsy was only performed in 1 patient, and the diagnosis of cerebral toxoplasmosis was confirmed.

Neurological coinfections were found in 9 (20%) patients. The etiological agents in these patients were cytomegalovirus (encephalitis: 3; polyradiculopathy: 2), *Treponema pallidum* (meningitis: 2), and 1 case each of *C. neoformans* and *M. tuberculosis* as meningoencephalitis. In addition to clinical and basic CSF characteristics and/or neuroradiological findings, CMV was diagnosed using CSF PCR. Research laboratory testing and fluorescent treponemal antibody absorption test on CSF were used to diagnose *T. pallidum* , and *C* . *neoformans* was diagnosed via cryptococcal antigen test, while *M* . *tuberculosis* , via CSF PCR.

### Treatment and outcomes

Trimethoprim-sulfamethoxazole was the initial anti- *T. gondii* agent in 43 (97%) patients, and pyrimethamine-sulfadiazine was preferred in 3 (7%) subjects. In total, 6 (13%) patients died during hospitalization, all of sepsis due to nosocomial pneumonia.

Patients with cerebral toxoplasmosis had a median length of hospital stay of 30 (IQR: 18–51) days, 8 patients (18%) were admitted to the ICU, and 6 patients (13%) died during hospitalization ( Table 1 ). Altered levels of consciousness ( *p* < 0.0001) and ICU admission ( *p* < 0.0001) were related to death ( Table 2 ). In the binary logistic regression, altered level of consciousness (odds ratio [OR]: 37; 95% confidence interval (95%CI): 2.8–474; *p* = 0.006) and ICU admission (OR: 42; 95%CI: 2.9–416; *p* = 0.002) were associated with in-hospital mortality, while neurological coinfection showed a trend towards significant association (OR: 5.3; 95%CI: 0.86–32.99; *p* = 0.072).

**Table 2:** Risk factors related to the death of 44 patients living with HIV/AIDS and cerebral toxoplasmosis

Variables	Survival (n = 38)	Death (n = 6)	*p*
Male sex: n (%)	18 (47)	4 (66)	0.380
Age in years: median (IQR)	44 (36–50)	40 (20–50)	0.628
CD4+ T lymphocytes in cells/mm ^3^ : median (IQR)	52 (18–98)	20 (8–76)	0.170
CD4+ T lymphocytes > 50 cell/mm ^3^ : n (%)	21 (55)	1 (16)	0.079
Time of cART failure in months: median (IQR)	28 (13–51)	19 (0–62)	0.493
History of cART: n (%)	34 (89)	5 (83)	0.660
Prophylaxis use: n (%)	23 (60)	4 (66)	0.774
cART failure > 1 year: n(%)	30 (78)	4 (66)	0.505
Duration of symptoms before admission in days: median (IQR)	9.5 (5–26)	12 (4–23)	0.881
Altered level of consciousness: n (%)	**7 (18%)**	**6 (100%)**	**< 0.0001**
Length of hospit stay in days: median (IQR)	29 (17–48)	47 (27–89)	0.113
ICU admission: n (%)	**4 (10)**	**5 (83)**	**< 0.0001**
Neurological coinfections: n (%)	6 (15)	3 (50)	0.054

Abbreviations: cART, combined antiretroviral therapy; ICU, Intensive Care Unit; IQR, interquartile range.

Note: Significant results appear in bold.

Patients with neurological coinfections had longer hospitalizations (26 days versus 62 days; *p* = 0.021) and were admitted more frequently to the ICU (14% versus 44%; *p* = 0.045) than the group without coinfections ( Table 3 ). An analysis of the CSFs was available for 29 (65%) patients, and th results differed only in that higher protein levels were found in the neurological coinfection group (53 mg/dL versus 154 mg/dL; *p* = 0.04).

**Table 3:** Comparative analysis of cerebral toxoplasmosis patients living with HIV/AIDS with and without neurological coinfection

Variables	Patients without neurological coinfection (n = 35)	Patients with neurological coinfection (n = 9)	*p*
Male sex: n (%)	16 (45)	6 (66)	0.262
Age in years: median (IQR)	44 (36–51)	45 (30–48)	0.878
CD4+ T lymphocytes in cells/mm ^3^ : median (IQR)	51 (16–98)	47 (8–102)	0.455
Time of cART failure in months: median (IQR)	25 (13–50)	22 (8–56)	0.250
History of cART: n (%)	32 (91)	7 (78)	0.242
Prophylaxis use: n (%)	23 (66)	4 (44)	0.242
Duration of symptoms before admission in days: median (IQR)	9 (5–25)	14 (6–34)	0.878
Altered level of consciousness: n (%)	9 (25)	4 (44)	0.272
Length of hospital stay in days: median (IQR)	**26 (15–45)**	**62 (30–75)**	**0.021**
ICU, admission: n (%)	**5 (14)**	**4 (44)**	**0.045**
Protease inhibitor: n (%)	12 (34)	3 (33)	0.957
Integrase Inhibitor: n (%)	4 (11)	9 (100)	0.287
CSF cells: median (IQR)	3.5 (2–15.5)	18 (2–68)	0.253
CSF protein: median (IQR)	54 (31–133)	154 (39–245)	0.04
CSF glucose: median (IQR)	60 (47–85)	51 (36–73)	0.199

Abbreviations: cART, combined antiretroviral therapy; CSF, cerebrospinal fluid; ICU, Intensive Care Unit; IQR, interquartile range.

Note: Significant results appear in bold.

## DISCUSSION

In the present study, 20% of the PLWHA with cerebral toxoplasmosis had neurological coinfections, and CMV was the most common etiology. Patients with neurological coinfections had longer hospital stays and a higher rate of ICU admissions.

The profile of the patients in the present study highlights the gap between public health system infrastructure (that is, a well-structured national program including universal and free distribution of cART) and its effectiveness. Most patients in the present study had been previously diagnosed with HIV/AIDS, had received cART and prophylaxis, and had had prior opportunistic diseases. Despite the fact that almost 90% of patients are cART-experienced, barriers to retention in care and regular use of therapy indicate a loss of opportunities in this vulnerable population.

Neurological coinfections in PLWHA are challenging and usually considered uncommon. In the clinical practice, the diagnosis is commonly delayed or unnoticed. Detailed neurological evaluations on admission and during hospitalization are key to enable the timely suspicion of neurological co-infection. For example, 1 patient with advanced HIV infection showed hemiparesis and decreased visual acuity upon admission, concomitant with cerebral toxoplasmosis and CMV retinitis. Another patient with advanced HIV infection was admitted due to headache and seizures, but developed worsening headache, nausea, and vomiting after a week of anti- *T. gondii* treatment. He was subsequently diagnosed with cerebral toxoplasmosis and cryptococcal meningitis. Seminal neuropathological postmortem studies have shown the importance of neurological co-infections in PLWHA. Lang et al. 6 demonstrated that 14% of their patients had neurological coinfections, and Jellinger et al. 7 found a 20% coinfection rate. It is plausible that neurological coinfections are misdiagnosed in the clinical practice, which can affect the outcomes of PLWHA. Few clinical studies have been conducted on concomitant neurological infections. Variables such as the rate of exposure to pathogens in the general population and availability of appropriate diagnostic tools may explain, in part, the presence of coinfections. Yang et al. 8 evaluated CSF samples using multiplex PCR and found a coinfection rate of 24%; *Cryptococcus* spp. and CMV were the two most common etiologies in the coinfection group. Siddiqi et al. 9 also evaluated multiple pathogens in CSF samples and found a 21% coinfection rate. In this study, 9 the main copathogens were *C.* spp. and the Epstein-Barr virus. Here, 20% of the patients with cerebral toxoplasmosis had neurological coinfections, and CMV was the most common pathogen. In all of these cases, molecular identification associated with neurological syndromes confirmed the presence of a concomitant neurological disease caused by CMV. This finding differs from the possibility of a non-pathogenic concomitant virus presence in the CSF. 9

Interestingly, the main coinfection with cerebral toxoplasmosis was caused by CMV. We have often observed this in patients admitted to our institution: CMV was the third most common pathogen associated with symptomatic neuroinfection in a general neurological cohort. 5 Our patients presented with approximately a decade of HIV infection diagnosis before admission and often had previous irregular use of cART, highlighting a prolonged and advanced HIV disease. Immune pathways regulate CMV reactivation, such as the Th1 response triggered by presentation of antigens through major histocompatibility complex-II (MHC-II) to CD4+ helper T cells, 10 and cytotoxic CD8+ T cell response. 11 Nevertheless, both of them are impaired in prolonged HIV infection. 11 Therefore, it is expected that patients will present with a high prevalence of CMV infections and end-organ diseases, not only in the neurological AIDS scenario but also in other clinical syndromes, as previously reported by our group. 12 Finally, the use of molecular diagnostic resources was fully available at our institution, and its importance has been demonstrated in CMV infection antemortem diagnoses, which were only established by CMV culture and biopsy. 13 14

The identification of concomitant neurological infections in PLWHA has important consequences at both the individual and public health levels. In the present study, patients with more than one neurological infection showed a trend towards higher in-hospital mortality rates. Nevertheless, this result may be attributed to the small sample size. Despite this limitation, our results show an association between neurological coinfections and longer hospitalization and/or ICU admissions. The management of HIV/AIDS has a high cost to the public health system compared to that of other chronic diseases. 15 Longer hospitalizations and more frequent ICU admissions in PLWHA for concomitant neurological infections increase this cost burden, which is particularly complicated in economically-constrained settings.

In the present study, the mortality rate was of 13%. The factors related to poor prognosis included ICU admission and altered levels of consciousness upon hospital admission. The PLWHA admitted to hospital facilities experience variable outcomes according to the setting. Li et al. 16 reported a mortality rate of 11% in China, which is similar to our results. Interestingly, they developed a prognosis score based on seven characteristics: fever, dizziness, memory deficits, disorders of consciousness, time of symptoms, CD4+ T cell count, and patchy lesion presence, with an accuracy of 91.1%. Nevertheless, Luma et al. 17 found a 30% mortality in Cameroon with the major factors relating to death including altered level of consciousness, focal signs, neck stiffness, or low CD4+ T cell count. Soneville et al. 18 evaluated patients admitted to the ICU only due to cerebral toxoplasmosis and found a mortality rate of 24%; Glasgow Coma Score and low CD4+ T cell count were related to poor prognosis. Despite the epidemiological and resource divergences among these studies, altered levels of consciousness were consistently associated with outcomes in patients with cerebral toxoplasmosis.

The present study has some limitations. First, only one center was included, with a relatively low number of patients. However, detailed prospective clinical and laboratory evaluations were performed. Second, the outcomes were evaluated exclusively during hospitalization; therefore, a long-term evaluation was impossible. However, the prolonged periods of hospitalization and follow-up strengthen our results. Finally, most patients were diagnosed with probable cerebral toxoplasmosis, a category commonly used in the clinical practice. Few patients had molecular confirmation of cerebral toxoplasmosis in their CSF samples, and the resultant interval between the initiation of anti- *T. gondii* treatment and CSF collection was prolonged. This delay may be partially due, to the contraindication of lumbar puncture CSF collection secondary to mass effect at hospital admission and routine practices at the time the study was carried out, using clinical and radiological parameters for the management of cerebral toxoplasmosis.

In conclusion, neurological coinfections were common in PLWHA with cerebral toxoplasmosis, and CMV was the main copathogen. The group of PLWHA with neurological coinfections had longer hospitalizations and a higher rate of ICU admissions. Coinfection is likely associated with increased mortality; however, further investigation is necessary. Our findings highlight the impact of neurological coinfections and their potential implications in the management and outcome of cerebral toxoplasmosis in PLWHA.
